# The association of FGF-21 with the risk of newly diagnosed type-2 diabetes mellitus: a cross-sectional study in Southern China

**DOI:** 10.1186/s12902-023-01426-y

**Published:** 2023-09-01

**Authors:** Lili You, Xiaosi Hong, Hongshi Wu, Diefei Liang, Feng Li, Dinghao Zheng, Xiuwei Zhang, Dan Liu, Qingyu Chen, Li Yan, Meng Ren, Wei Wang

**Affiliations:** 1 Department of Endocrinology, Sun Yat-sen Memorial Hospital, Sun Yat-sen University, No.107 Yan Jiang West Road, Guangzhou, 510120 China; 2 Guang Dong Clinical Research Center for Metabolic Diseases, Guangzhou, People’s Republic of China; 3 Department of Endocrinology, Dongguan People’s Hospital, Dongguan, People’s Republic of China; 4 Department of Medical Examination Center, Sun Yat-sen Memorial Hospital, Sun Yat-sen University, Guangzhou, People’s Republic of China; 5 Department of Endocrinology, Shenshan Medical Center, Sun Yat-sen Memorial Hospital, Sun Yat-sen University, Guangzhou, People’s Republic of China

**Keywords:** Type 2 diabetes mellitus, Fibroblast growth factor 21, Biomarker, Risk assessment model

## Abstract

**Background:**

This study investigated the relationship between fibroblast growth factor 21 (FGF-21) and newly diagnosed type-2 diabetes mellitus (T2DM).

**Methods:**

In this cross-sectional study, FGF-21 and T2DM risk were analyzed using restricted cubic splines with univariate or multivariate logistic regression analysis. Odds ratios (ORs) and 95% confidence intervals (CIs) were calculated via logistic regression analysis. Cluster and subgroup analyses were conducted to evaluate the associations between FGF-21 and diabetes in different subpopulations. Nomograms and ROC curves were used to explore the clinical utility of FGF-21 in the diabetes assessment model.

**Results:**

High levels of FGF-21 were significantly associated with a high risk of T2DM after adjusting for confounding factors in both the total population and subpopulations (P for trend < 0.001). In the total population, the ORs of diabetes with increasing FGF-21 quartiles were 1.00 (reference), 1.24 (95% CI 0.56–2.80; quartile 2), 2.47 (95% CI 1.18–5.33; quartile 3), and 3.24 (95% CI 1.53–7.14; quartile 4) in Model 4 (P < 0.001), and the trend was consistent in different subpopulations. In addition, compared with the model constructed with conventional noninvasive indicators, the AUC of the model constructed by adding FGF-21 was increased from 0.668 (95% CI: 0.602–0.733) to 0.715 (95% CI: 0.654–0.777), indicating that FGF-21 could significantly improve the risk-assessment efficiency of type-2 diabetes.

**Conclusion:**

This study demonstrated that a high level of circulating FGF-21 was positively correlated with diabetes, and levels of FGF-21 could be an important biomarker for the assessment of diabetes risk.

## Introduction

Type-2 diabetes mellitus (T2DM) is the world’s most common chronic metabolic disease, and it is associated with major economic and social costs [1, 2]. According to the latest statistics published in 2021, the 10th edition of the International Diabetes Federation Diabetes Atlas showed that the number of patients with diabetes around the world is 463 million and is predicted to reach 700 million by 2045 [3]. Globally, approximately 12.2% of deaths are caused by diabetes or its complications [3]. Thus, it is extremely important to explore biomarkers related to the occurrence and progression of diabetes to provide a basis for the prevention, diagnosis and treatment of diabetes in the clinic.

The 22 members of the fibroblast growth factor (FGF) family are responsible for multiple biological functions, including the growth and development of cells, the formation of angiogenesis, and the healing of wounds [4–6]. In animal models, it has been shown that FGF-21, which is mostly expressed in liver and muscle tissue, can significantly improve glucose and lipid metabolism as well as overall energy balance ^7–9^. Moreover, in cross-sectional studies, it has also been shown that FGF-21 concentration is elevated in patients suffering from obesity, T2DM, metabolic syndrome, and nonalcoholic fatty liver disease [10–13]. In addition, a prospective study conducted in China over 5.4 years revealed significant changes in plasma FGF-21 levels in subjects with prediabetes (pre-DM) and T2DM as well as predicting the onset of diabetes [14]. The association between FGF-21 and T2DM and its related metabolic diseases indicated that FGF-21 may be a promising biomarker and antidiabetic target, which has attracted the attention of researchers. However, drug therapy and disease course may be confounding factors in diabetes patients enrolled in previous studies, and the association between FGF-21 and newly diagnosed T2DM is still lacking in southern China.

Accordingly, this study aimed to determine the stability of the association between FGF-21 and the risk of newly diagnosed T2DM in different populations and evaluated the efficiency of FGF-21 in diabetes risk assessment. Our findings could provide a foundation for the use of FGF-21 as a promising biomarker for the early diagnosis and treatment of T2DM.

## Materials & methods

### Study population

**Fig. 1:**
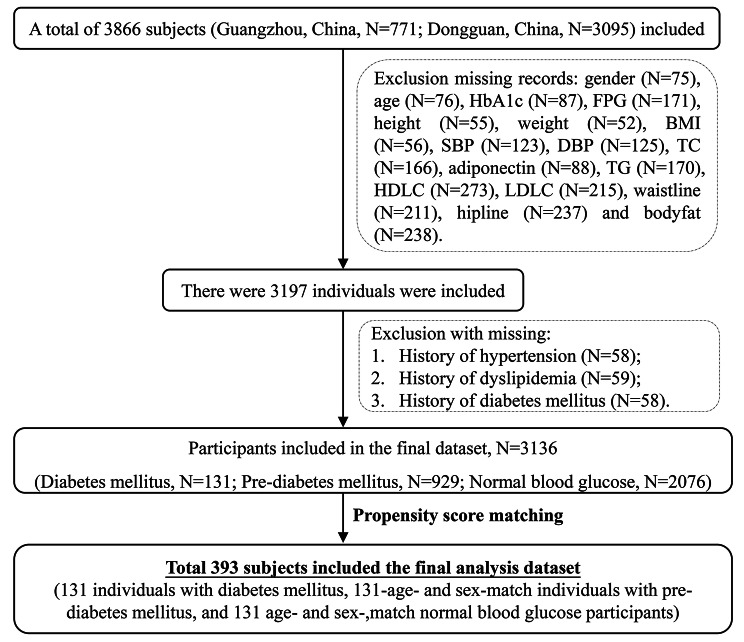
Flow chart of the study design

The present study was a cross-sectional study conducted in Guangzhou and Dongguan, China, between December 2018 and October 2019. In this study, participants were recruited from communities in Guangzhou (Sun Yat-sen Memorial Hospital) and Dongguan (Dalingshan community, Zhangmu community, Daojiao community, Qiaotou community, Songshan lake community, Qingxi community, Zhang’an community, and Meinian Physical Examination Center). The inclusion criteria are subjects between the ages of 18 and 70, the Han ethnic Chinese population, and permanent residents living (≥ 3 years). The exclusion criteria were as follows: [1] pregnancy; [2] self-reported mental illness or severe physical diseases, such as hepatic cirrhosis, chronic renal failure, or evident cardiac insufficiency; [3] self-reported infectious disease or malignant tumors; [4] self-reported medical history of hypertension or dyslipidemia at the recruitment stage of the study (to eliminate the potential influence of blood pressure and lipid-lowering medications on the distribution of FGF21 in the body); [5] self-reported other endocrine diseases; and [6] long-term use of drugs, dietary supplements or functional food (≥ 3 times/week for more than 3 months). This study consisted of 669 participants who did not provide basic clinical characteristics information, and 61 participants who did not provide information regarding a history of hypertension, dyslipidemia, or diabetes were excluded. A total of 3136 subjects were included, including 131 subjects with diabetes, 929 subjects with prediabetes, and 2076 subjects with normal blood glucose. Propensity score matching (PSM) is a statistical methodology that is used to deal with data derived from observational studies to reduce variation between groups and to make them more comparable. As a final step, data analysis was carried out on 131 subjects with diabetes, 131 age- and sex-matched subjects with prediabetes, and 131 age- and sex-matched subjects with normal blood glucose. An overview of the process is shown in the flow diagram (Fig. 1). The Ethics Committee of Sun Yat-sen Memorial Hospital approved this study ([2019] No. 38). Informed written consent was obtained from all individuals.

### Measurements

Anthropometric measurements were performed by trained staff following standard protocols. Height and weight were recorded separately, and the units were accurate to 0.1 cm and 0.1 kg, respectively. Participants were required to remove their shoes and wear light indoor clothing during the measurement. The body mass index (BMI) was calculated by dividing the weight of the individual in kilograms by the height of the individual in meters squared (kg/m^2^). With a measurement accuracy of 0.1 cm, the waistline was measured at the umbilicus, and the hipline was measured at the widest point over the great trochanters at the level of the umbilicus. The systolic blood pressure (SBP) and diastolic blood pressure (DBP) were measured over at least 5 min with automatic electronic equipment (OMRON, Omron Company, Japan), and the average value was used for subsequent analysis after measuring the blood pressure twice with electronic equipment.

Venous blood samples were collected after overnight fasting for at least 10 h, centrifuged, and kept at -80 °C until the assays were performed at the end of the day. With the assistance of an automatic biochemical analyzer (Beckman, USA), fasting plasma glucose (FPG) levels, total cholesterol (TC), triglycerides (TG), low-density lipoprotein cholesterol (LDL-C) and high-density lipoprotein cholesterol (HDL-C) levels were measured. High-performance liquid chromatography (Bio-Rad, USA) was used to measure glycosylated hemoglobin and type A1C (HbA1c) levels. Enzyme-linked immunosorbent assay (ELISA) for FGF-21 (catalog number: ab222506) was conducted with the aid of an automatic biochemical analyzer (Mindray, China). The intra- and interassay coefficients of variation were 5.3% and 5.5%, respectively.

### Definitions

According to the American Diabetes Association diagnostic criteria, diabetes is defined as FPG ≥ 7.0 mmol/L or HbA1C ≥ 6.5%, and prediabetes is defined as 6.1 mmol/L ≤ FPG < 7.0 mmol/L or 5.7 ≤ HbA1c < 6.5% [15]. Hypertension was diagnosed by systolic blood pressure (SBP) ≥ 140 mmHg or diastolic blood pressure (DBP) ≥ 90 mmHg [16]. According to the World Health Organization (WHO) criteria, overweight/obesity is defined as BMI ≥ 25 kg/m2 [17]. However, compared to Caucasians, Chinese individuals have higher percentages of body fat, cardiovascular risk factors, and overall mortality rates at the same BMI level [18]. Therefore, considering that our study population consisted of Chinese individuals, we also defined overweight/obesity as BMI ≥ 24 kg/m2 [18, 19]. According to WHO criteria, central obesity is defined as WC ≥ 80 cm for females or ≥ 90 cm for males [17]. The diagnosis of dyslipidemia was based on the presence of one or more of the following criteria: total cholesterol (TC) ≥ 5.20 mmol/L, triglycerides (TG) ≥ 1.70 mmol/L, high-density lipoprotein cholesterol (HDL-C) < 1.00 mmol/L or low-density lipoprotein cholesterol (LDL-C) ≥ 3.4 mmol/L[20].

### Cluster analysis

The variables of cluster analysis were selected based on their clinical relevance and the representativeness of disease features associated with each disease type. BMI, waistline, SBP, TGs, and FPG were chosen for cluster analysis. To identify which clusters were most appropriate for the present study, the optimal cluster was determined using NbClust () of the NbClust package in RStudio, and two clusters were identified as the most appropriate cluster when using the cluster () function. To obtain the subgroups, we performed a K-means clustering analysis, and the k-value for this analysis was set to 2 using the Kmeans () function of the stats package in RStudio to perform a K-means clustering analysis.

### Statistical analysis

Continuous variables with a normal distribution are presented as the mean ± standard deviation (SD). To test the differences between the groups, one-way ANOVA was used on all data, and post hoc comparisons were performed using the Bonferroni correction. Continuous variables that did not follow normal distribution were expressed as medians (interquartile ranges), and differences among groups were evaluated using the Kruskal‒Wallis test. For categorical variables, the results are expressed as numbers (proportions). Categorical variables were compared using the χ^2^ test. The shape of the dose‒response relationship between FGF-21 and the odds ratio of diabetes was visualized using restricted cubic splines. Odds ratios (ORs) and their corresponding 95% confidence intervals (95% CIs) were calculated using unadjusted and multivariate-adjusted logistic regression analyses. Receiver operating characteristic (ROC) curves were plotted to investigate the role of FGF-21 in assessing the risk of newly diagnosed T2DM. Two diabetes risk assessment models were constructed as follows: Model 1 (including the noninvasive factors of sex, age, BMI, waistline, and SBP) and Model 2 (including sex, age, BMI, waistline, SBP, and FGF-21). Accordingly, nomograms were established based on Model 2. To verify the stability of the results, forest plots based on subgroup analysis results were further performed, which were stratified as follows: male or female, age ≤ 55 or age >55, sex, age, hypertension or not, overweight and obesity or not, central obesity or not, dyslipidemia or not. All statistical analyses were performed using R-Studio version 3.6.1 software. Statistical tests were two-sided, and a P value of < 0.05 was considered to indicate statistical significance.

## Results

### Clinical characteristics of the study population

In this study, 393 subjects were included in the analysis dataset by propensity score matching. The clinical characteristics of the study population are shown in Table 1. Among the 393 subjects included in the present study, 131 subjects had T2DM, 131 age- and sex-matched subjects had pre-DM, and 131 subjects were age- and sex-matched subjects with normoglycemia. Compared with the nondiabetes and prediabetes groups, the diabetes group had higher levels of weight, BMI, WC, HC, WHR, SBP, DBP, TGs, TC, FPG, and HbA1c (all P for trend < 0.05), supporting that the population with worse glycemic status tended to have poorer metabolic profiles. In addition, an increasing trend of FGF-21 in the normoglycemia group (146.6 [95.5, 247.3] pg/mL), prediabetes group (160.6 [104.3, 246.3] pg/mL), and diabetes group (257.8 [159.8, 339.2] pg/mL) was detected in this study (P for trend < 0.001).

**Table 1:** Baseline characteristics of study population according to glucometabolic state

Variables	Normoglycemia	Pre-diabetes	Diabetes	P _difference_	P _trend_
Male, %	63 (52.07)	63 (51.22)	57 (53.77)	0.927	0.806
Age, years	53.83 ± 11.26	53.56 ± 11.55	53.68 ± 11.13	0.983	0.917
Height, cm	160.06 ± 8.55	160.24 ± 9.02	161.43 ± 10.29	0.489	0.275
Weight, kg	59.91 ± 10.93	63.46 ± 11.09	67.88 ± 13.54^*#^	< 0.001	< 0.001
BMI, kg/m^2^	23.25 ± 3.05	24.66 ± 3.51^*^	25.95 ± 4.02^*#^	< 0.001	< 0.001
Waistline, cm	81.52 ± 8.97	84.68 ± 7.91^*^	88.50 ± 11.01^*#^	< 0.001	< 0.001
Hipline, cm	93.90 ± 6.67	95.62 ± 5.85	98.08 ± 7.76^*#^	< 0.001	< 0.001
WHR	0.87 ± 0.06	0.89 ± 0.06	0.90 ± 0.07^*^	< 0.001	< 0.001
SBP, mmHg	121.57 ± 12.83	123.41 ± 12.77	126.37 ± 19.94^*^	0.062	0.020
DBP, mmHg	73.75 ± 8.47	74.99 ± 8.03	78.29 ± 12.08^*#^	0.001	< 0.001
TC, mmol/L	5.26 ± 0.92	5.46 ± 0.99	5.57 ± 1.10	0.070	0.023
TG, mmol/L	1.16 (0.85, 1.62)	1.32 (0.93, 1.92)	1.67 (1.22, 2.52)	< 0.001	< 0.001
HDL-C, mmol/L	1.46 ± 0.39	1.49 ± 0.45	1.49 ± 0.73	0.864	0.622
LDL-C, mmol/L	3.19 ± 0.74	3.29 ± 0.81	3.28 ± 0.96	0.664	0.462
FPG, mmol/L	4.77 ± 0.43	5.29 ± 0.63^*^	7.70 ± 2.85^*#^	< 0.001	< 0.001
HbA1c, %	5.29 ± 0.27	5.82 ± 0.32^*^	7.27 ± 1.69^*#^	< 0.001	< 0.001
FGF-21, pg/mL	146.6 (95.5, 247.3)	160.6 (104.3, 246.3)	257.8 (159.8, 339.2) ^*^	0.004	< 0.001

1. Data are expressed as mean ± SD or medians (interquartile ranges) for skewed variables or numbers (proportions) for categorical variables

2. TG and FGF-21 were skewed in distribution, log transformation before variance analysis and comparison in pairs

3. P values were for the χ2 analysis or analysis of variance across the groups.

4. ^*^P < 0.05 compared with normoglycemia population; ^#^P < 0.05 compared with pre-diabetes.

5. BMI, body mass index; WHR, waistline-hipline ratio; SBP, systolic blood pressure; DBP, diastolic blood pressure; TC, total cholesterol; TG, triglycerides; HDL-C, high-density lipoprotein cholesterol; LDL-C, low-density lipoprotein cholesterol; FPG, fasting plasma glucose; HbA1c, glycosylated hemoglobin, type A1C; FGF-21, fibroblast growth factor 21.

In Table 2, participants’ characteristics are shown according to their FGF-21 quartiles. FGF-21 quartiles were positively associated with diabetes indicators (FPG), obesity indicators (BMI, WC, HC, and WHR), hypertension indicators (SBP and DBP) and dyslipidemia indicators (TG) (all P for trend < 0.05).

**Table 2:** Clinical characteristics of participants according to quartiles of FGF-21

Variables	Quartile 1	Quartile 2	Quartile 3	Quartile 4	P _difference_	P _trend_
Male, %	52 (59.09)	45 (51.72)	42 (48.28)	44 (50.00)	0.496	0.197
Age, years	53.35 ± 11.58	51.69 ± 11.17	55.52 ± 11.08	54.19 ± 11.17	0.242	0.153
Height, cm	161.61 ± 7.09	160.68 ± 9.41	160.19 ± 9.54	159.68 ± 10.70	0.557	0.155
Weight, kg	61.62 ± 11.25	63.40 ± 13.83	63.24 ± 10.32	66.03 ± 12.99	0.118	0.025
BMI, kg/m^2^	23.48 ± 3.25	24.39 ± 4.05	24.59 ± 3.17	25.79 ± 3.86^*^	< 0.001	< 0.001
Waistline, cm	82.00 ± 9.43	84.18 ± 10.60	84.86 ± 7.59	87.93 ± 10.08^*#^	< 0.001	< 0.001
Hipline, cm	94.09 ± 6.36	95.06 ± 7.67	95.91 ± 4.94	98.02 ± 7.90^*#^	0.001	< 0.001
WHR	0.87 ± 0.06	0.88 ± 0.07	0.88 ± 0.05	0.90 ± 0.06^*^	0.052	0.009
SBP, mmHg	121.93 ± 14.25	121.84 ± 14.18	122.75 ± 13.87	128.14 ± 18.20^*#^	0.017	0.008
DBP, mmHg	74.16 ± 9.14	74.59 ± 8.83	76.06 ± 9.89	77.44 ± 10.73	0.101	0.015
TC, mmol/L	5.31 ± 0.86	5.45 ± 1.00	5.26 ± 0.95	5.67 ± 1.15^@^	0.033	0.065
TG, mmol/L	1.04 (0.75, 1.50)	1.28 (0.91, 1.81) ^*^	1.43 (1.16, 1.99) ^*^	1.76 (1.24, 3.09) ^*#@^	< 0.001	< 0.001
HDL-C, mmol/L	1.44 ± 0.31	1.52 ± 0.43	1.51 ± 0.67	1.46 ± 0.64	0.683	0.882
LDL-C, mmol/L	3.27 ± 0.69	3.27 ± 0.84	3.11 ± 0.86	3.36 ± 0.92	0.251	0.792
FPG, mmol/L	5.65 ± 2.13	5.47 ± 1.55	5.84 ± 1.97	6.38 ± 2.38^#^	0.020	0.008
HbA1c, %	5.97 ± 1.48	6.02 ± 1.44	6.08 ± 0.94	6.24 ± 1.11	0.511	0.144

^*^P < 0.05 compared with quartile 1; ^#^P < 0.05 compared with quartile 2; ^@^ P < 0.05 compared with quartile 3.

### The association of FGF-21 with DM risk

**Fig. 2:**
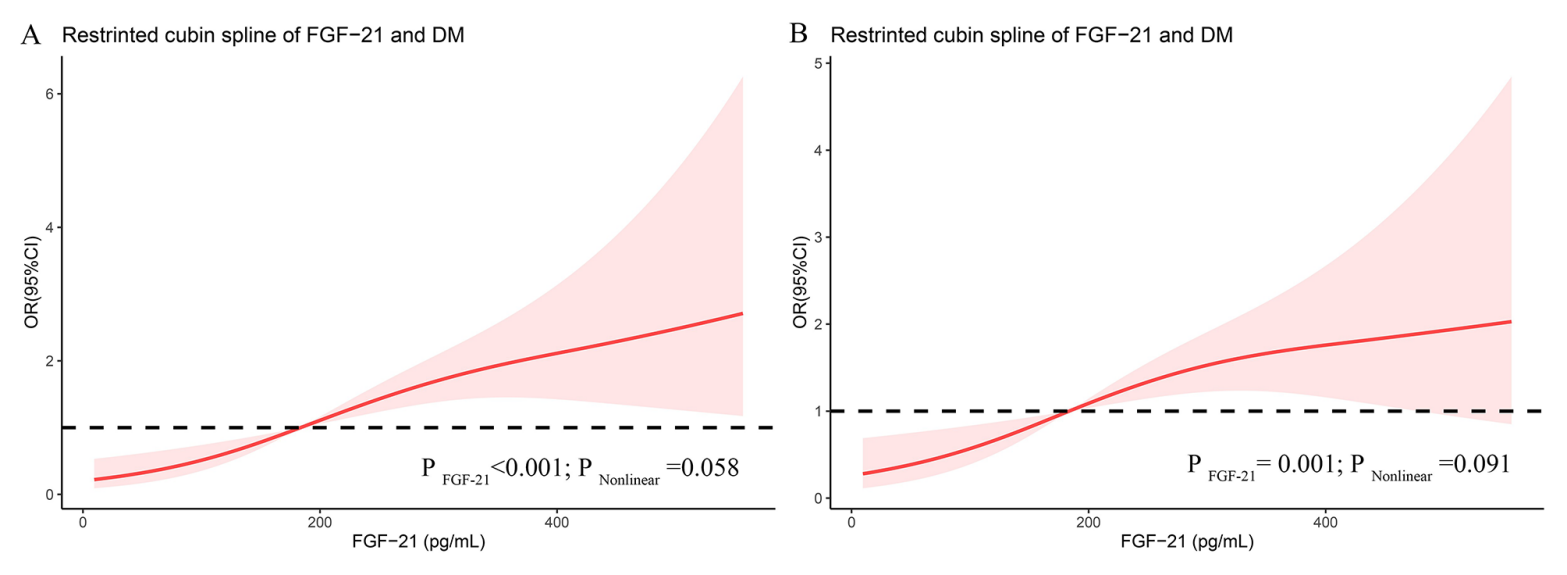
Dose-response association of FGF-21 with risk of diabetes (**A**) Univariate regression analysis; (**B**) Multivariate logistic regression analysis adjusted for gender, age, BMI, WC, SBP and TG Odds ratios (OR) are indicated by solid lines and 95% CIs by shaded areas

Subsequently, restricted cubic splines were used to show the possibility of nonlinear relationships between FGF-21 and diabetes. As shown in Fig. 2A, univariate regression analysis indicated that a higher level of FGF-21 was significantly associated with higher odds of diabetes (P for FGF-21 < 0.001; P for nonlinear = 0.058). After adjusting for sex, age, BMI, WC, SBP, and TGs, the linear association between FGF-21 and diabetes was also confirmed using multivariate logistic regression analysis (P for FGF-21 = 0.001; P for nonlinear = 0.091), suggesting that a curvilinear association between FGF-21 and the risk of newly diagnosed type-2 diabetes did not exist (Fig. 2B).

### Cluster analysis

**Fig. 3:**
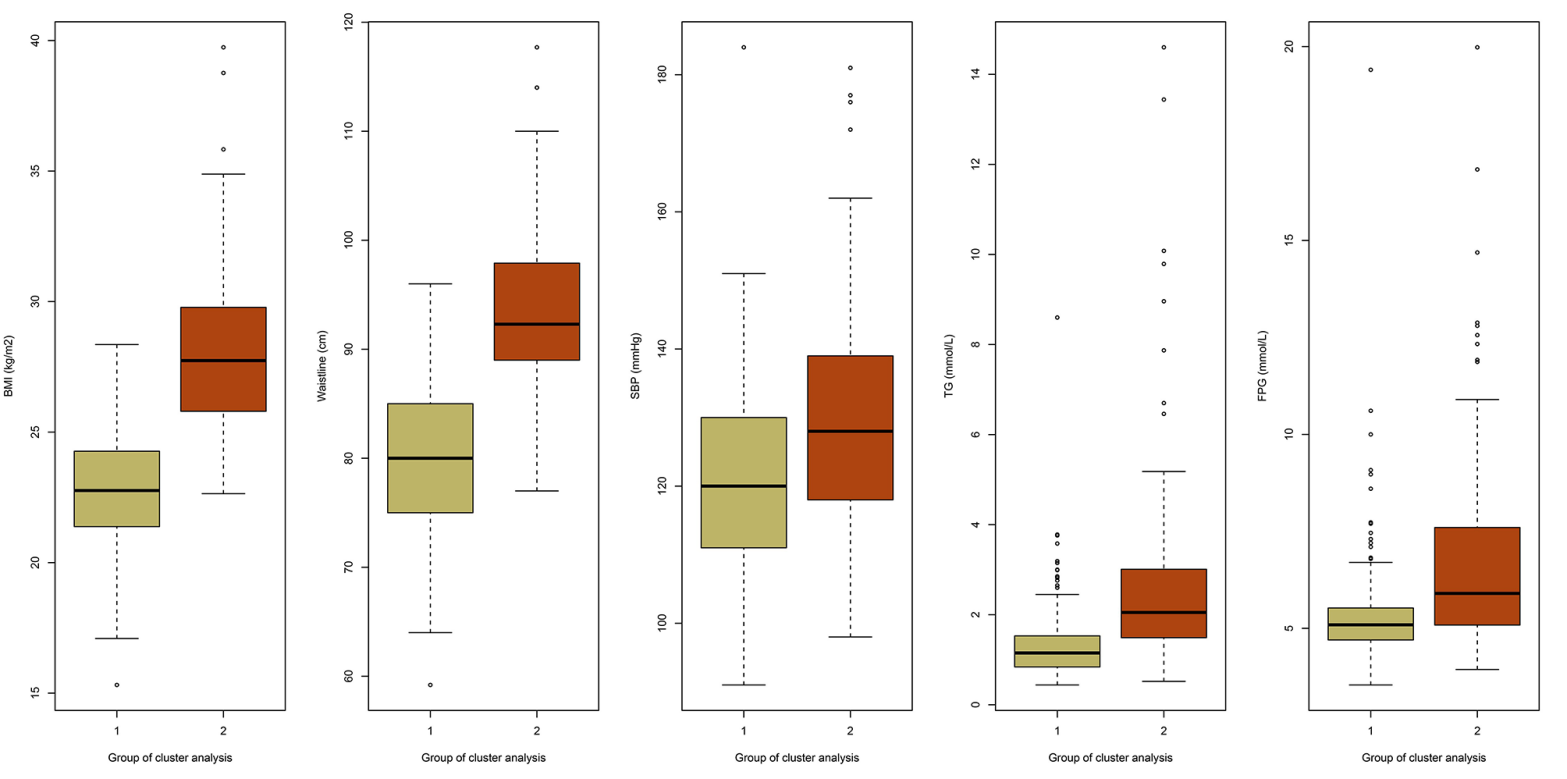
Cluster characteristics of participants Distributions of BMI, waistline, SBP, TG, and FPG at registration, for each cluster. Two clusters were identified and colored in the histogram: green, data for cluster 1; red, data for cluster 2

In the cluster analysis, BMI, waistline, SBP, TGs, and FPG were chosen to divide the total population into subpopulations. Accordingly, participants were classified into two subgroups (Fig. 3). Cluster 1, termed the relatively healthy population, was characterized by relatively low BMI, waistline, SBP, TGs, and FPG. Cluster 2, labeled ‘metabolic derangement subjects’, was characterized by a higher BMI, waistline, SBP, TGs, and FPG. Specifically, the basic clinical characteristics according to the clusters are presented in Table 3. Additionally, it is particularly worth noting that Cluster 2 demonstrated a higher level of FGF-21 than that of Cluster 1 (152.6 [98.6, 244.4] vs. 247.7 [146.3, 339.5] pg/mL, P < 0.05).

**Table 3:** Clinical characteristics of participants in different clusters

Variables	Cluster 1	Cluster 2	P _difference_
Male, %	107 (47.13)	76 (61.79)	0.012
Age, years	54.26 ± 11.42	52.64 ± 11.03	0.202
Height, cm	159.89 ± 8.45	161.74 ± 10.53	0.076
Weight, kg	58.24 ± 8.96	73.41 ± 11.32	< 0.001
BMI, kg/m^2^	22.69 ± 2.34	28.02 ± 3.16	< 0.001
Waistline, cm	79.87 ± 6.73	93.74 ± 7.68	< 0.001
Hipline, cm	92.86 ± 5.32	101.15 ± 6.37	< 0.001
WHR	0.86 ± 0.05	0.93 ± 0.06	< 0.001
SBP, mmHg	120.16 ± 13.10	130.15 ± 17.17	< 0.001
DBP, mmHg	73.05 ± 8.55	80.20 ± 10.08	< 0.001
TC, mmol/L	5.32 ± 0.92	5.61 ± 1.13	0.010
TG, mmol/L	1.15 (0.84, 1.53)	2.05 (1.49, 3.01)	< 0.001
HDL-C, mmol/L	1.53 ± 0.46	1.38 ± 0.63	0.012
LDL-C, mmol/L	3.21 ± 0.75	3.34 ± 0.96	0.166
FPG, mmol/L	5.30 ± 1.37	6.82 ± 3.66	< 0.001
HbA1c, %	5.82 ± 1.05	6.55 ± 1.48	< 0.001
FGF-21, pg/mL	152.6 (98.6, 244.4)	247.7 (146.3, 339.5)	0.005

Cluster 1, termed as the relatively healthy population, was characterized by relatively low BMI, waistline, SBP, TG, and FPG;

Cluster 2, labeled as metabolic derangement subjects, was characterized by higher level of BMI, waistline, SBP, TG, and FPG.

### The relationship between FGF-21 and newly diagnosed T2DM

The associations of FGF-21 in quartiles with the odds of diabetes are shown in Table 4. Initially, in the total population, FGF-21 was consistently associated with diabetes after adjustment for confounding factors, including sex, age, BMI, waistline, SBP, and TGs. The ORs of newly diagnosed T2DM with increasing FGF-21 quartiles were 1.00 (reference), 1.24 (95% CI 0.56–2.80; quartile 2), 2.47 (95% CI 1.18–5.33; quartile 3), and 3.24 (95% CI 1.53–7.14; quartile 4) in Model 4 (P < 0.001). Likewise, the positive association between FGF-21 and diabetes was also represented in different cluster populations.

**Table 4:** Adjusted odds ratios for newly diagnosed diabetes by quartiles of FGF-21 (Compared with pre-diabetes and normoglycemia population)

Models	Quartile 1	Quartile 2	Quartile 3	Quartile 4	P _trend_
Total population					
Model 1	1.00	1.48(0.69–3.22)	2.93(1.45–6.16)	4.83(2.43–10.07)	< 0.001
Model 2	1.00	1.49(0.69–3.26)	3.00(1.48–6.33)	4.92(2.47–10.29)	< 0.001
Model 3	1.00	1.30(0.59–2.90)	2.60(1.25–5.59)	3.64(1.77–7.82)	< 0.001
Model 4	1.00	1.24(0.56–2.80)	2.47(1.18–5.33)	3.24(1.53–7.14)	< 0.001
Cluster 1 population					
Model 1	1.00	2.35(0.83–7.22)	4.65(1.77–13.81)	2.67(0.86–8.73)	0.022
Model 2	1.00	2.33(0.83–7.17)	4.56(1.72–13.61)	2.63(0.84–8.64)	0.025
Model 3	1.00	2.46(0.86–7.67)	4.99(1.84–15.27)	2.78(0.88–9.27)	0.022
Model 4	1.00	2.36(0.82–7.40)	4.60(1.66–14.33)	2.56(0.79–8.69)	0.044
Cluster 2 population					
Model 1	1.00	0.56(0.16–1.94)	1.03(0.32–3.37)	3.04(1.00-9.58)	0.005
Model 2	1.00	0.54(0.15–1.92)	1.05(0.32–3.48)	3.09(1.00-9.92)	0.005
Model 3	1.00	0.51(0.13–1.95)	1.22(0.36–4.19)	3.19(1.00-10.60)	0.004
Model 4	1.00	0.53(0.13–2.05)	1.26(0.37–4.35)	3.53(1.05–12.48)	0.004

Model 1 is unadjusted;

Model 2 is adjusted for sex and age;

Model 3 is adjusted for sex, age, BMI, waistline, and SBP;

Model 4 is adjusted for sex, age, BMI, waistline, SBP, and TG.

### ROC curves and nomogram

**Fig. 4:**
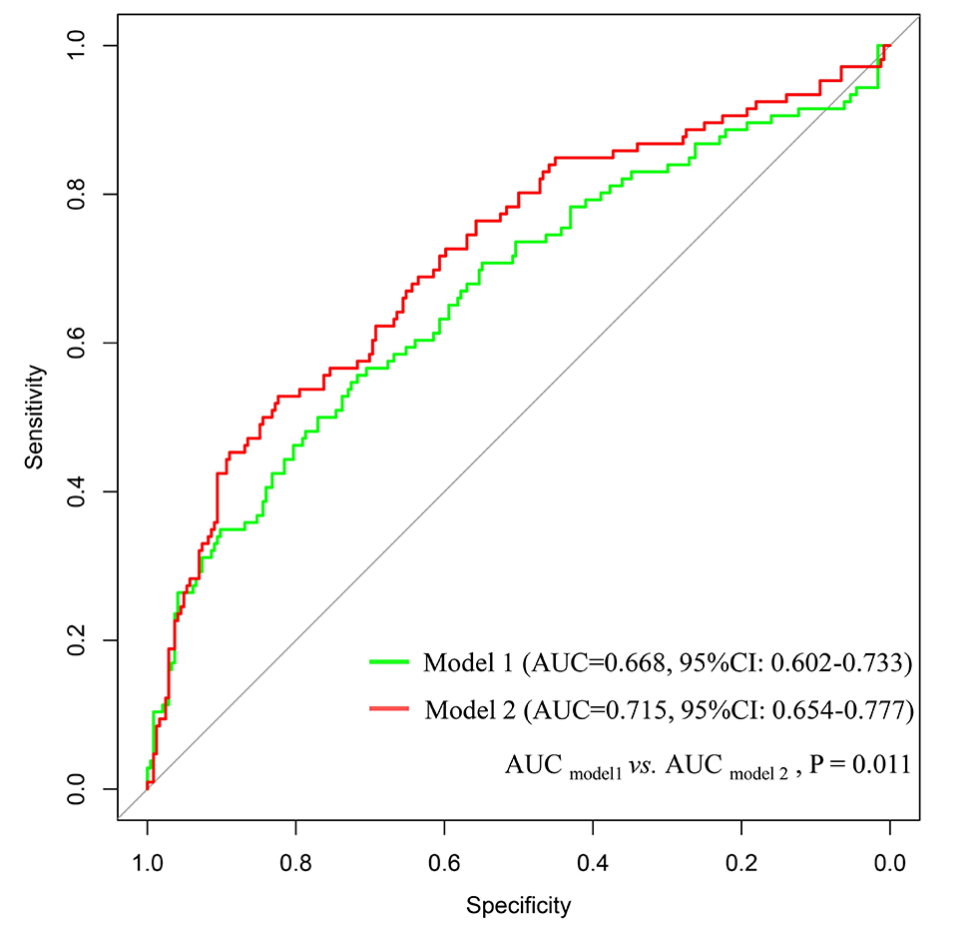
ROC curve of the association between adiponectin and newly diagnosed diabetes (**A**) Green curve, Model 1 including the non-invasive factors of sex, age, BMI, waistline, and SBP; (**B**) Model 2 including sex, age, BMI, waistline, SBP, and FGF-21

**Fig. 5:**
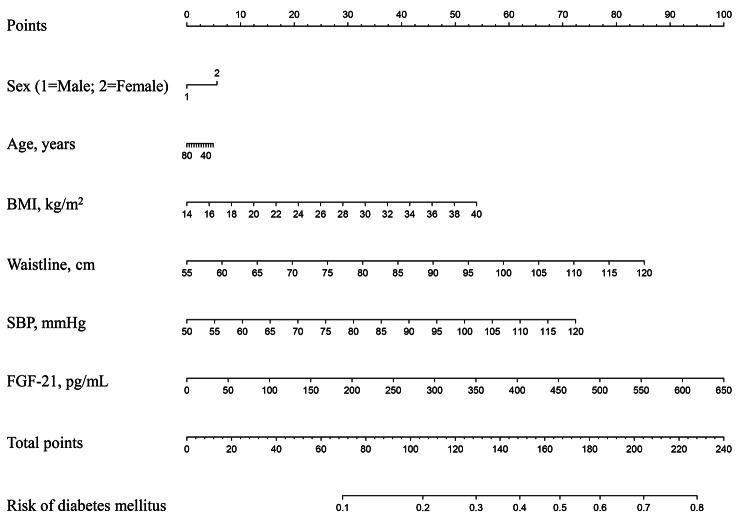
Factors influencing diabetes in the multivariate logistic analysis in the nomogram model According to the clinical and anthropometric characteristics of the subjects, the total points were counted. The diabetes rate was matched with total points

Accordingly, we constructed the following two diabetes risk assessment models: Model 1 (including the noninvasive factors of sex, age, BMI, waistline, and SBP) and Model 2 (including sex, age, BMI, waistline, SBP, and FGF-21). Compared with Model 1 (area under curve (AUC) = 0.668 [95% CI: 0.602–0.733]), the AUC of Model 2 constructed by adding invasive screening factors of FGF-21 was significantly increased to 0.715 [95% CI: 0.654–0.777]), indicating that FGF-21 could significantly improve the evaluation efficiency of diabetes risk (Fig. 4). Therefore, we applied Model 2 (including noninvasive factors and FGF-21) to construct a nomogram, and the model was presented as the nomogram to evaluate the risk of diabetes (Fig. 5).

### Subgroup and sensitivity analysis

**Fig. 6:**
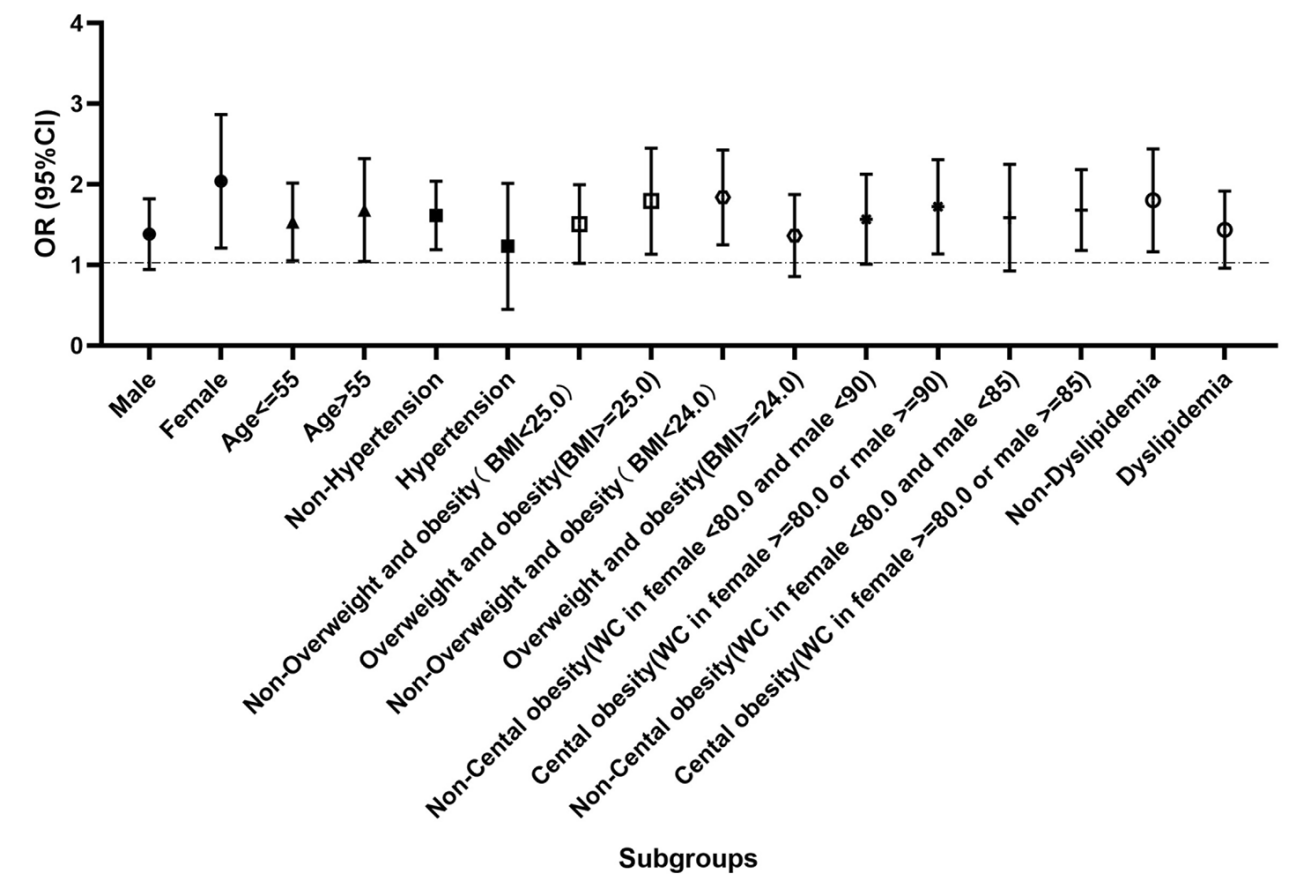
The association of FGF-21 with newly diagnosed diabetes in different subgroups

Subgroup analysis was conducted to evaluate the sensitivity of the observed association between FGF-21 and newly diagnosed T2DM. As shown in the corresponding forest plot in Fig. 6, high levels of FGF-21 were associated with a higher risk of newly diagnosed T2DM in different subgroups. Specifically, a significant relationship of FGF-21 level with increased odds of diabetes was detected in women, younger, and older subjects, subjects without hypertension, subjects without overweight and obesity, central obesity subjects and nondyslipidemia subjects (all P < 0.05).

## Discussion

In this cross-sectional study, our research group strengthened the evidence that increased FGF-21 was significantly associated with the risk of newly diagnosed T2DM. Even after adjusting for conventional risk factors, such as sex, age, BMI, waistline, SBP, and TGs, the association continued to exist in the total population and in different subpopulations. It was also demonstrated that FGF-21 provided additional value in the construction of the diabetes assessment model when combined with conventional noninvasive factors.

Since FGF-21 was first identified in mouse embryos in 2000, an increasing number of studies have shown a positive relationship between FGF-21 and various metabolic diseases in humans [7]. High circulating levels of FGF-21 are found in patients suffering from obesity-related disease [10, 11, 13, 14]. In agreement with previous studies, FGF-21 quartiles were positively correlated with diabetes indicators (FPG), obesity indicators (BMI, WC, HC and WHR), hypertension indicators (SBP and DBP) and dyslipidemia indicators (TG) in this study.

Subsequently, this study demonstrated that FGF-21 was positively related to the risk of diabetes in the total population and subpopulation using restricted cubic splines and multiple logistic regression analyses. Similar to our studies, a meta-analysis, including 11 studies with 866 patients with T2DM and 629 controls, suggested that patients with T2DM have significantly higher plasma FGF-21 levels, and FGF-21 levels are influenced by BMI, TC and TG [21]. Meanwhile, Chen et al. noted that plasma FGF-21 levels are affected independently by fasting blood glucose, plasma insulin, and insulin resistance assessed by a homeostasis model, further supporting that FGF-21 may be involved in the pathogenesis of insulin resistance and diabetes [22]. However, due to ethnic or regional differences, the results of this study also need to be verified in different regions and ethnic groups. Notably, our study population was newly diagnosed with type-2 diabetes without the intervention of drugs and other therapeutic factors, which makes the association analysis of FGF-21 and diabetes more definite. Accordingly, our study used a variety of statistical approaches to confirm the current gap in the association of FGF-21 with newly diagnosed type-2 diabetes in the southern China region, providing evidence for the prevention, diagnosis and treatment of diabetes in the clinic.

Studies have identified FGF-21 as an independent risk factor for metabolic syndrome and diabetes. Further analyses were performed to estimate the practical value of FGF-21 combined with noninvasive factors of sex, age, BMI, waistline, and SBP. Compared with Model 1, which included the noninvasive factors of sex, age, BMI, waistline, and SBP, Model 2, which added FGF-21, significantly increased the AUC from 0.668 (95% CI: 0.602–0.733) to 0.715 (95% CI: 0.654–0.777) (P = 0.011), demonstrating the value of FGF-21 in assessing the risk of diabetes. In a recent study that investigated the performance of FGF-21 in diabetes prediction, relative to other adipokines and established risk factors including 2-hour plasma glucose (2hG) during the oral glucose tolerance test (OGTT), it was also found that FGF-21 performed a superior prediction value in the construction of a diabetes risk assessment model [23]. Specifically, compared with the diabetes prediction model (DP) based on age, family history, smoking, hypertension, BMI, dyslipidemia, and FPG, the AUC of the “DP + FGF- 21” model increased from 0.797 to 0.819 (P = 0.0072), rendering its performance compared to the “DP + 2hG” model (AUC = 0.838) [23]. In addition, a number of studies have reported that FGF-21 could be an independent predictor of fatty liver, chronic liver failure, coronary heart disease, and prognosis in patients with diabetes [24–30]. In general, our study identified the important value of FGF21 in the risk assessment of newly diagnosed type-2 diabetes in southern Chinese populations. Furthermore, except for FGF21, our model only included noninvasive indicators, which do not add additional invasive burden to the patients. Since most information can be obtained by questionnaires and measurements, our model could be worthy of popularization and application. Therefore, it is important to develop FGF-21 as a routine examination index for disease prediction and prognostic evaluation of multiple diseases in the clinic.

To the best of our knowledge, this is the first study focusing on the association of FGF-21 with newly diagnosed T2DM in Southern China. Initially, participants with self-reported diabetes were excluded during the recruitment phase, which avoids the confounding effect of antidiabetic drugs on glycemic control. Meanwhile, we could reduce confounding by the duration of diabetes by including patients who were newly diagnosed with T2DM. Finally, this study strengthened the ability of FGF-21 in diabetes risk assessment in the total population and subpopulation with the use of multiple statistical approaches, including multivariate regression analysis, cluster analysis and subgroup analysis. In summary, the present findings further confirm the relationship between high levels of FGF-21 and diabetes, indicating the potential ability of FGF-21 as a biomarker for the risk assessment of diabetes. At the same time, our results also provide evidence to investigate the mechanism of FGF-21 and its relationship with the development of type-2 diabetes mellitus.

However, several limitations should be taken into account in this study. First, this cross-sectional study was unable to establish any causal relationships between FGF-21 and the development of type-2 diabetes. Second, it is undetermined whether our results can be generalized to other ethnic groups, as this study focused exclusively on Guangdong community residents in China. Furthermore, although we adjusted for a variety of covariates associated with diabetes in the multivariate regression analyses, we should have adjusted for other potential mediators, such as exercise, diet, and lifestyle, as well as incomplete data collection, since they could have potentially resulted in residual confounding. For further validation of the clinical application of FGF-21 as a risk assessment indicator in the development of diabetes, prospective cohort studies with larger sample sizes are necessary. Despite the limitations described above, the current study showed favorable application prospects and provided a solid basis for further research.

## Conclusion

In summary, this study revealed that FGF-21 was consistently positively correlated with the risk of newly diagnosed T2DM in the total population and in subpopulations in southern China. FGF-21 could be used as a biomarker in diabetes risk assessment. This clinical study might provide a foundation for the pathogenesis, early diagnosis, and effective treatment of diabetes.

## Data Availability

The datasets used and/or analyzed during the current study available from the corresponding author on reasonable request.

## Abbreviations

T2DM: Type 2 diabetes mellitus
BMI: Body mass index
WHR: Waistline-hipline ratio
SBP: Systolic blood pressure
DBP: Diastolic blood pressure
TC: Total cholesterol
TG: Triglycerides
HDL-C: High-density lipoprotein cholesterol
LDL-C: Low-density lipoprotein cholesterol
FPG: Fasting plasma glucose
HbA1c: Glycosylated hemoglobin, type A1C
FGF-21: Fibroblast growth factor 21
PSM: Propensity score matching
OR: Odds ratio
95% CI: 95% confidence interval
ROC: Receiver operating characteristic
2hG: 2-hour plasma glucose
OGTT: Oral glucose tolerance test
